# Nucleolar stress in C9orf72 and sporadic ALS spinal motor neurons precedes TDP-43 mislocalization

**DOI:** 10.1186/s40478-021-01125-6

**Published:** 2021-02-15

**Authors:** Olubankole Aladesuyi Arogundade, Sandra Nguyen, Ringo Leung, Danielle Wainio, Maria Rodriguez, John Ravits

**Affiliations:** University of California, San Diego, La Jolla, CA USA

## Abstract

Nucleolar stress has been implicated in the pathology and disease pathogenesis of amyotrophic lateral sclerosis (ALS) and frontotemporal lobar degeneration (FTLD) from repeat expansions of GGGGCC in C9orf72 (C9-ALS/FTLD) but not in sporadic ALS (SALS). Previously we reported that antisense RNA transcripts are unique in C9-ALS because of their nucleolar localization in spinal motor neurons and correlation with TDP-43 mislocalization, the hallmark proteinopathy of ALS and FTLD. Here we report our further studies of 11 SALS, 11 C9-ALS and 11 control spinal cords. We find that nucleolar stress manifests specifically as shrinkage in nucleoli of C9-ALS spinal motor neurons. Nucleolar size reduction is greatest in similarly sized alpha motor neurons from C9-ALS cases and results are not skewed by the number of surviving neurons from each ALS spinal cord. Surprisingly, nucleolar shrinkage occurs before main pathological hallmarks—TDP-43 mislocalization or antisense RNA foci—appear and this suggest that nucleolar stress can precede pathology in C9-ALS, findings previously identified in C9-FTLD using sense RNA foci and dipeptide repeat proteins as pathological markers. Importantly, these observations are also seen in SALS motor neurons and thus nucleolar stress appears to be a significant and probably upstream problem in sporadic disease.

## Introduction

Nucleolar stress has been implicated in C9orf72 amyotrophic lateral sclerosis (ALS) and frontotemporal dementia (FTD), but this has not been specifically studied in sporadic ALS (SALS) [1, 16, 26, 36, 39, 58]. Normally, humans express 2–20 copies of the C9orf72 intronic repeat of GGGGCC, but the repeat can be expanded to thousands of copies. Toxicity is produced from repeat expansion-containing RNA transcripts that sequester RNA binding proteins and are exported to the cytoplasm, producing repeat-associated non-ATG translated dipeptide repeat proteins (DPRs) [11, 42]. The repeat expansion is bidirectionally transcribed to produce sense and antisense RNA transcripts. DPRs containing the arginine residue, sense-encoded poly(GR) and antisense-encoded poly(PR), have repeatedly been found to be most toxic when overexpressed in cells and animal models [3, 9, 14, 22, 23, 27, 32, 35, 40, 46, 52–54, 58–64, 68]. In cellular models, poly(GR) and poly(PR) co-localize to nucleoli and cause nucleolar stress characterized by alterations of nucleolar size and impaired ribosomal biogenesis [23, 26, 58]. However, poly(GR) and poly(PR) were not found to co-localize to nucleoli in patient tissue [46].

Overexpression of sense RNA transcripts causes sequestration of nucleolin and disruption of ribosomal biogenesis [16, 31]. Both sense and antisense RNA transcripts form perinucleolar RNA foci in patient cells [1, 10]. Compared to sense RNA foci, antisense RNA foci localize to the nucleolus with increased prevalence in brain and spinal cord regions that undergo neurodegeneration [1]. Nucleolar antisense RNA foci are increased in frontal cortical neurons of C9-ALS patients diagnosed with FTD and correlate with elevated TDP-43 pathology, the hallmark proteinopathy of ALS [1]. In spinal motor neurons, antisense RNA foci and the presence of nucleolar antisense RNA foci correlate with TDP-43 mislocalization, suggesting that nucleolar antisense RNA foci are relevant to disease pathogenesis [1, 10].

Nucleolar stress has been reported in C9 frontotemporal lobar degeneration (FTLD) pathology. Overall, C9-FTLD frontal cortical neurons had smaller nucleoli when compared to controls. However, neurons with sense RNA foci, poly(GR) or poly(GA) had larger nucleoli than neurons without these pathological features. It remains unknown what accounts for the smaller nucleolar size and it is possible that TDP-43 mislocalization, antisense RNA foci or other pathological markers correlate with decreased nucleolar size. Nucleolar size alterations have also been observed in C9-ALS patient lymphocytes, fibroblasts and patient derived iPS neurons [16]. In these cellular models, nucleoli were found to be larger when compared to controls and this phenotype was recapitulated by overexpressing 21 repeats of GGGGCC in HEK293T cells [16]. Nucleolin staining was dispersed from C9 lymphocytes and fibroblast nucleoli and scattered in the nucleus [16]. Interestingly, overexpression of poly(GR) in HeLa cells competitively sequesters ribosomal RNA and nucleophosmin and causes the dispersion of the nucleolus [59]. These cellular phenotypes were specific to C9-ALS compared to controls, non-ALS or non-C9-ALS. RNA profiling of C9-ALS iPS cells revealed that ribosomal biogenesis is a main pathway dysregulated [19]. Similarly, decreases in ribosomal maturation have been reported in C9-ALS lymphoblasts and motor cortex [16]. Alterations to nucleolar size in ALS indicate nucleolar stress is an important nexus in disease pathogenesis [16, 26, 36, 54, 58, 59].

The nucleolus is a membraneless organelle composed of RNA, DNA and proteins. The dynamic structure of the nucleolus is intrinsically linked to its multiple roles in cellular physiology. By convention the primary function of the nucleolus is ribosomal biogenesis but beyond this, the nucleolus facilitates multiple processes including cellular stress response, genome maintenance and repair, cell cycle progression, development and aging [4, 30, 37, 41]. In this study, we sought to determine if alterations of nucleolar size occur in C9-ALS spinal motor neurons and to compare them to SALS. We aimed to elucidate whether nucleolar stress is specific for C9-ALS or also occurs in SALS and additionally to determine how alterations of nucleolar size might correlate with key pathological markers—TDP-43 mislocalization and antisense RNA foci. Surprisingly, we found that nucleolar size is as reduced in sporadic ALS as in C9-ALS and that shrinkage occurs even in the absence of pathological markers, thus suggesting nucleolar dysfunction precedes TDP-43 mislocalization.

## Materials and methods

### CNS tissues

Human tissues were obtained using a short-postmortem interval acquisition protocol. The ALS nervous systems were from patients who presented with ALS as the clinical phenotype, with or without FTD, and all had progressed and died from their motor impairment. For this study, we evaluated 11 C9-ALS cases (confirmation by repeat-primed PCR and southern blotting) and 11 SALS cases.

### CNS region and cell types

In spinal cord, we examined the alpha and gamma motor neurons in Rexed lamina IX of anterior horn lumbosacral sections. Alpha motor neurons were classified by their large multipolar cytoplasm, presence of lipofuscin and a single prominent nucleolus. Estrogen related receptor gamma (ESRRG) immunostaining was used to classify gamma motor neurons.

### Immunohistochemistry (IHC)

On day 1, sections were deparaffinized with Citrisolv (Fisher Scientific #04-355-121) and hydrated with different dilutions of alcohol. Endogenous peroxidase activity was quenched with 0.06% H_2_O_2_ for 15 min. Antigen retrieval was performed in a high pH solution (Vector #H-3301) in a pressure cooker for 20 min at 120 °C. Following antigen retrieval, sections were permeabilized with 1% FBS (Atlanta Biologicals #511150) and 0.2% Triton X-100 (Sigma #65H2616) in PBS for 15 min and then blocked with 1% FBS in PBS for 25 min. The sections were incubated overnight with the primary antibody, rabbit polyclonal anti-ESRRG (1:500, ProteinTech, Cat# 14017–1-AP). On the second day, after 60 min incubation with the secondary antibody (Immpress reagent kit, anti-rabbit, Vector) at room temperature, signals were detected using Immpact DAB (Vector #sk-4105) for 1–5 min. Counterstaining was performed with 0.1% Cresyl Ecth Violet. For IHC visualization, slides were scanned with Hamamatsu Nanozoomer 2.0HT Slide Scanner at 40 × magnification. We analyzed 2 8 µm sections per patient.

### Immunofluorescence (IF)

On day one, sections were deparaffinized with Citrisolv (FISHER brand #04-355-121) and hydrated through a serial dilution of ethanol. Sections were permeabilized with 1% FBS (Atlanta Biologicals #511150) and 0.2% Triton X-100 (Sigma #65H2616). Following permeabilization, antigen retrieval was performed in a high pH solution (Vector # H- 3301) in a pressure cooker for 20 min at 120 °C. Next, sections were blocked with 2% FBS in 1 × PBS for 60 min and were incubated with primary antibody overnight. Primary antibodies were diluted in 2% FBS in 1X PBS. For TDP-43, we used mouse monoclonal anti-TDP-43 (NovusBiologicals, # H00023435-M01, 1/500) and for ESRRG we used rabbit polyclonal anti-ESRRG (1:500, ProteinTech, Cat# 14017-1-AP). On day two, slides were incubated with secondary antibodies (goat anti-mouse Abcam #1150116, Alexa594, 1/500 and donkey anti-rabbit Jackson Immuno Research #715545, Alexa 488, 1/500) for 60 min at room temperature. Secondary antibodies were diluted in 2% FBS in 1X PBS. We quenched CNS auto-fluorescence with 0.1% Sudan Black in 70% ethanol for 15 s. Slides were cover slipped using ProLong Gold Antifade Mountant with DAPI. We analyzed 2 8 µm sections per patient.

### Co-fluorescence in situ hybridization (co-FISH-IF)

Sections were deparaffinized and permeabilized as stated above. We next performed prehybridization and hybridization with FISH probes (GGGGCC3-Cy3, 80 nM, ISIS, #693839) as described in [1] and incubated the slides overnight at 66 °C. On day two, slides were washed three times with 2 × SSC for 20 min at 55 °C and then washed twice in 0.2 × SSC for 20 min at 55 °C. Next, sections were blocked with 2% FBS in 1 × PBS for 60 min and were incubated with primary antibody overnight. Primary antibodies were diluted in 2% FBS in 1X PBS. For TDP-43, we used rabbit polyclonal anti-TDP-43 (ProteinTech, #12892–1-AP, 1/1000). For fibrillarin, we used mouse monoclonal anti-fibrillarin (SC-166001, 1/100). On day three, slides were incubated with secondary antibodies (donkey anti-mouse Jackson Immuno Research #715545, Alexa 488, 1/500 and goat anti-rabbit Abcam #31634, Alexa 647, 1/500) for 60 min at room temperature. Secondary antibodies were diluted in 2% FBS in 1X PBS. We quenched CNS auto-fluorescence with 0.1% Sudan Black in 70% ethanol for 15 s. Slides were cover slipped using ProLong Gold Antifade Mountant with DAPI. We analyzed 2–4 8 µm sections per patient.

### Confocal microscopy

For IF and co-FISH-IF, neurons were visualized using the fast mode for Zeiss 800 laser scanning microscope with airyscan, under 40X water magnification. Neurons were only quantified if the nucleus of a neuron was present in the imaged plane. Maximum projections of z-stacks were compiled using Zen Black. Nucleolar, nuclear and cytoplasmic area were calculated using Image J. The number and intracellular localization of antisense RNA foci and the presence of TDP-43 in the nucleus or mislocalized to the cytoplasm were observed. Quantifications were made by a single observer in a blinded manner.

### Statistics

Variables were compared using two-tailed Mann–Whitney tests and Kolmogorov–Smirnov tests. Error bars are presented as standard error of mean and are presented centered on the arithmetic mean. *p* values below 0.05 were considered significant. All data analysis and graphing were performed using custom Python scripts and GraphPad Prism version 8.2.1 for Windows (GraphPad Software, La Jolla, CA).

## Results

### Nucleolar size is reduced in C9-ALS and SALS spinal motor neurons

**Fig. 1:**
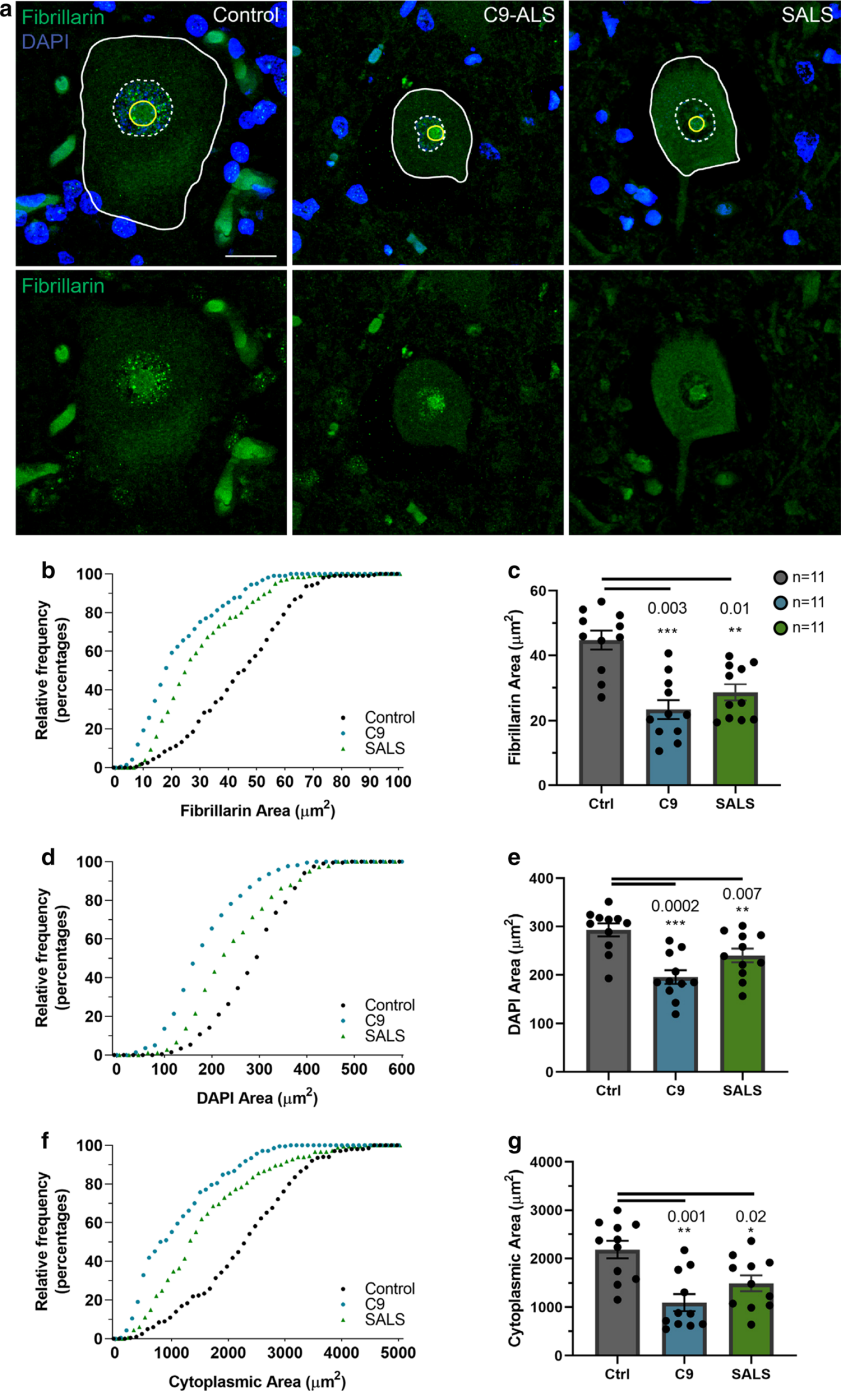
Nucleolar shrinkage occurs in C9-ALS and SALS spinal motor neurons. **a** Nucleolar area is identified by fibrillarin staining (green), nuclear area is identified by DAPI and cytoplasmic area is identified by autofluorescence in control, C9-ALS and SALS spinal motor neurons. **b**–**g** Average nucleolar (**b**, **c**), nuclear (**d**, **e**) and cytoplasmic area (**f**, **g**) were decreased in C9-ALS and SALS cases compared to controls

We measured nucleolar, nuclear and cytoplasmic areas using fibrillarin immunofluorescence, DAPI immunofluorescence and background fluorescence respectively in lumbosacral spinal motor neurons (SMNs) from 11 SALS, C9-ALS and control nervous systems each (Table 1, Fig. 1a and Additional file 2: Fig. S2a). All three compartments were smaller in C9-ALS and SALS than controls either when combined within groups or averaged within each nervous system (all *p* values < 0.02) (Fig. 1b–g). In order to determine whether the smallness was more specific to one compartment or another, we compared ratios of nucleolar to nuclear area, nucleolar to cytoplasmic area and nuclear to cytoplasmic area. Compared to controls, the ratio of nucleolar to nuclear area was significantly decreased in both C9-ALS and SALS SMNs (C9-ALS *p* = 0.003; SALS *p* = 0.007) (Additional file 1: Fig. S1a, b), indicating greater shrinkage of nucleoli compared to nuclei. Since the ratio of nuclear to cytoplasmic area was increased in both C9-ALS and SALS SMNs compared to controls (C9-ALS *p* = 0.01; SALS *p* = 0.09) (Additional file 1: Fig. S1e, f), indicating greater shrinkage of cytoplasm compared to nuclei, the ratio of nucleolar to cytoplasmic area was not an analogous comparison. Indeed, the ratios of nucleolar to cytoplasmic areas were slightly increased in C9-ALS or similar in SALS SMNs compared to controls (C9-ALS *p* = 0.1; SALS *p* > 0.9), consistent with greater shrinkage in cytoplasm (Additional file 1: Fig. S1c, d), a result that will be addressed later.

**Table 1:** Patient demographics (*DC* disease course, *DO* disease onset, *A* axial, *CI* cognitive impairment, *LL* lower limb, *S* swallowing)

Group	Case ID	Age (years)	Sex	DC (years)	DO (years)	Site of onset
Control	4	75	M	NA	NA	NA
Control	7	61	M	NA	NA	NA
Control	10	78	M	NA	NA	NA
Control	39	77	M	NA	NA	NA
Control	42	61	M	NA	NA	NA
Control	65	82	M	NA	NA	NA
Control	67	77	M	NA	NA	NA
Control	76	68	M	NA	NA	NA
Control	78	58	F	NA	NA	NA
Control	103	92	F	N/A	N/A	N/A
Control	115	94	M	N/A	N/A	N/A
C9-ALS	14	73	F	1.5	71.5	Bulbar
C9-ALS	81	58	M	1.5	56.5	Bulbar
C9-ALS	91	61	M	3.5	57.5	Bulbar
C9-ALS	120	64	M	7.5	56.5	Bulbar
C9-ALS	126	70	M	1.8	68.25	Arm
C9-ALS	119-JHU	61	F	2.6	58	LL
C9-ALS/FTD	86-JHU	74	M	7.3	67	Bulbar,S, LL, CI
C9-ALS/FTD	88-JHU	59	M	1.7	57	Bulbar, Speech, CI
C9-ALS	MN2	55	F	2.3	52	Bulbar, Speech
C9-ALS	PCJ	58	M	3.6	54	A, Respiratory, Trunk
C9-ALS	PYF	59	M	0.4	58	LL
SALS	29	77	F	3	74	LL
SALS	32	71	M	1.5	69.5	Respiratory, Trunk
SALS	43	74	M	1.8	72.25	Respiratory, Trunk
SALS	46	51	F	2.5	48.5	Arm
SALS	60	58	F	3	55	Bulbar
SALS	94	62	M	7	55	Bulbar
SALS	109	49	M	1.5	47.5	Leg
SALS	110	53	M	Unknown	N/A	Body twitches
SALS	111	59	F	3.5	55.5	Leg
SALS	123	57	M	4	53	Body twitches
SALS	125	67	M	1.5	65.5	Arm

In these determinations, there are differences in the number of surviving SMNs from each ALS nervous system, ranging between 9 and 45 neurons. In order to verify that our results were not skewed by variation in numbers of surviving neurons, we re-analyzed our data by randomly sampling neurons from each case, re-sampling and analyzing between 2 to 13 neurons for 1000 permutations (Additional file 2: Fig. S2a). As the sample size of randomly selected neurons increased from 2 to 13, the observed statistical significance stabilized at counting 6 neurons (Additional file 2: Fig. S2b–g), verifying our results were not biased by patho-anatomic sampling. Thus, we observe and validate overall size reduction in C9-ALS and SALS especially affecting the nucleolar subcellular compartment.

### Nucleolar size reduction is greatest in similarly sized C9-ALS alpha motor neurons

**Fig. 2:**
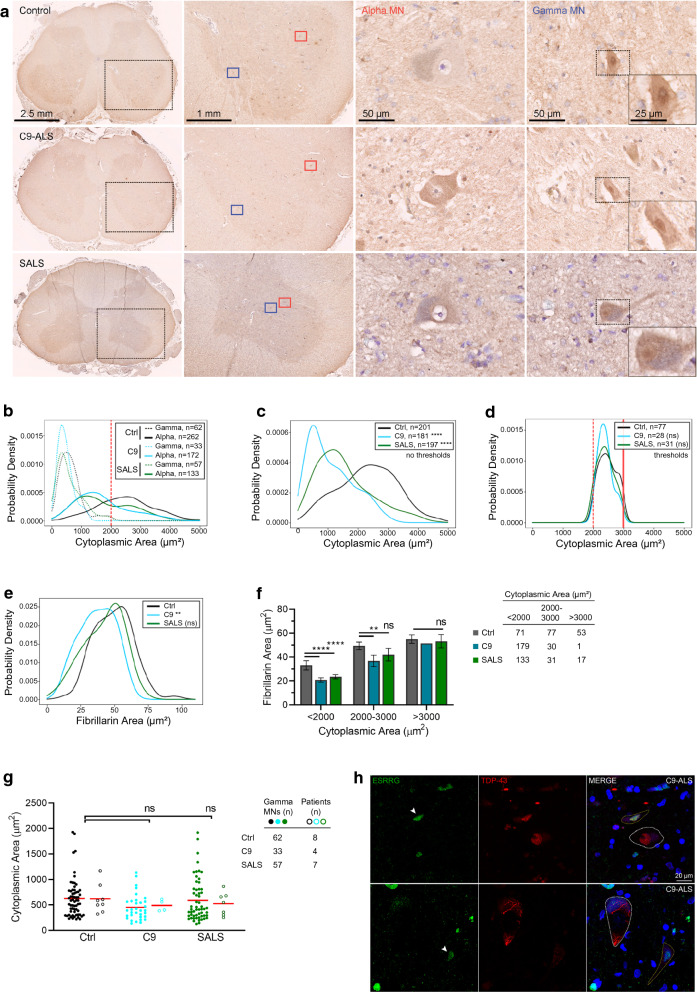
Gamma motor neurons are identified by ESRRG immunohistochemistry and immunofluorescence. **a** Immunohistochemistry of ESRRG in control, C9-ALS and SALS lumbar spinal cord with cresyl violet counterstain. Alpha motor neurons (third column) lack nuclear ESRRG immunofluorescence. Gamma motor neurons (fourth column) have nuclear ESRRG immunofluorescence. Magnification scales are consistent for each column. **b** Distribution of cytoplasmic area for gamma and alpha motor neurons in control, C9-ALS and SALS lumbar spinal cord (dashed line: threshold for alpha motor neurons). **c**–**f** Stratification of immunofluorescence data.** c** Distribution of cytoplasmic area from un-stratified immunofluorescence data. **d** Comparison of similar distribution of cytoplasmic area in sub-sample of control, C9-ALS and SALS alpha motor neurons (cytoplasmic area thresholds at 2000 µm^2^ and 3000 µm^2^). **e** Nucleolar area is decreased in sub-sample of C9-ALS neurons compared to control neurons with similar cytoplasmic size. **f** Nucleolar area in subsamples based on cytoplasmic size. **g** Distribution of cytoplasmic area in control, C9-ALS and SALS gamma motor neurons. **h** TDP-43 mislocalization occurs in alpha motor neurons (white dashed outline) and in gamma motor neurons (yellow dashed outline) identified by nuclear ESRRG immunofluorescence (arrowheads)

Since cytoplasmic area was decreased in both C9-ALS and SALS SMNs, we wondered if our analysis was specific for alpha motor neurons or skewed by a heterogeneous neuronal population including gamma motor neurons and whether or not there was a size bias because of overall shrinkage in ALS. Gamma motor neurons are small neurons that reside in Rexed lamina IX in the anterior part of anterior horns along with alpha motor neurons. In mice, expression of estrogen related receptor gamma (ESRRG), has been used to identify gamma motor neurons—gamma motor neurons have nuclear expression of ESRRG and alpha motor neurons do not [15]. We used ESRRG expression to identify gamma and alpha motor neurons in a subset of SALS (n = 7), C9-ALS (n = 6) and control (n = 8) nervous systems. Nuclear ESRRG was found in putative gamma motor neurons and was absent in all putative alpha motor neurons (Fig. 2a). As expected, gamma motor neurons were smaller than alpha motor neurons in control, C9-ALS and SALS cases (Fig. 2b). C9-ALS and SALS gamma motor neuron size was not significantly different compared to controls (*p* < 0.06, *p* < 0.09; KS-test) (Fig. 2g). On average, gamma motor neurons were 630 ± 370 µm^2^ in controls, 450 ± 250 µm^2^ in C9-ALS and 590 ± 420 µm^2^ in SALS (Fig. 2b, g). On average, alpha motor neurons were 2490 ± 960 µm^2^ in controls, 1690 ± 870 µm^2^ in C9-ALS and 1820 ± 970 µm^2^ in SALS (Fig. 2b). Based on our findings using immunohistochemistry, we determined a lower threshold of 2000 µm^2^ cytoplasmic area was sufficient to exclude potential gamma motor neurons from our immunofluorescence data assessing nucleolar size differences (Fig. 2b).

We sought to further establish whether nucleolar size decrease correlated with the overall size change of alpha motor neurons. In the original immunofluorescence data, C9-ALS and SALS alpha motor neurons were significantly smaller than controls (C9-ALS *p* < 0.0001, SALS *p* < 0.0001; KS-test). C9-ALS and SALS alpha motor neuron size was not significantly different from each other (*p* = 0.2) (Fig. 2c). To assess a sample of neurons that were of uniform size in control, C9-ALS and SALS cases, we used a lower threshold of 2000 µm^2^ and an upper threshold of 3000 µm^2^ (Fig. 2d). The lower threshold excluded (1) potential gamma motor neurons and (2) small putative alpha motor neurons in ALS, and the upper threshold further ensured uniform size (Additional file 3: Fig. S3a). Indeed, it appeared that the decrease in the nucleolar size occurred in the uniformly sized alpha motor neurons—this was statistically significant in C9-ALS and had a strong trend in SALS (C9-ALS *p* = 0.009, SALS *p* = 0.1) (Fig. 2e, f). Further, in these uniformly sized alpha motor neurons, the ratios of nucleolar to nuclear area and nucleolar to cytoplasmic area were also decreased in C9-ALS (*p* = 0.01, *p* = 0.02) and trended toward significance in SALS SMNs (*p* = 0.06, *p* = 0.1) (Additional file 3: Fig. S3b, c). Conversely, nuclear area and the ratio of nuclear to cytoplasmic area were similar to controls in both C9-ALS (*p* = 0.7, *p* = 0.9) and SALS SMNs (*p* = 0.8, *p* > 0.9) (Additional file 3: Fig. S3d, e). We additionally found that the decrease in nucleolar size was significantly reduced in SMNs smaller than 2000 µm^2^ (*p* < 0.0001) (Fig. 2f). This supports that the decreased nucleolar size that affects alpha motor neurons, the main targets of neurodegeneration, may be independent of overall size change not only in C9-ALS but likely also in SALS.

An important but not well-studied aspect of ALS pathology is the involvement of gamma motor neurons in TDP-43 proteinopathy. TDP-43 proteinopathy has been observed in non-alpha motor neuron cell types including oligodendrocytes and large neurons in Clarke’s column [5]. Since we were able to define gamma motor neurons with ESRRG immunostaining (Fig. 2a), we used this to examine if TDP-43 mislocalization also occurred in gamma motor neurons. Similar to alpha motor neurons lacking ESRRG expression, C9-ALS gamma motor neurons expressing nuclear ESRRG also had mislocalized nuclear TDP-43 that formed cytoplasmic inclusions (Fig. 2h). Interestingly, we did not observe this in SALS.

### Sex specific SMN size differences are disrupted in C9-ALS and SALS

We also wondered if differences in patient’s sex, age of disease onset or duration of disease correlated with changes in SMN nucleolar size. In our study, the majority of C9-ALS and SALS cases were male and were age and sex matched to controls (Table 1). We found that in controls, cytoplasmic and nuclear area were significantly larger in males compared to females (*p* = 0.04, *p* = 0.04) but nucleolar area was not significantly different when stratified by sex (*p* = 0.1) (Additional file 4: Fig. S4a, b, c). Sex differences were not present in C9-ALS SMNs when we compared cytoplasmic, nuclear or nucleolar area (*p* < 0.9; *p* = 0.5; *p* = 0.9, respectively) or in SALS SMNs (*p* = 0.2; *p* = 0.5; *p* = 0.4, respectively) (Additional file 4: Fig. S4a, b, c). There was no correlation between disease onset or disease course and SMN size indices (Additional file 4: Fig. S4d–i).

### Nucleolar size reduction occurs in neurons with and without antisense RNA foci in C9-ALS

**Fig. 3:**
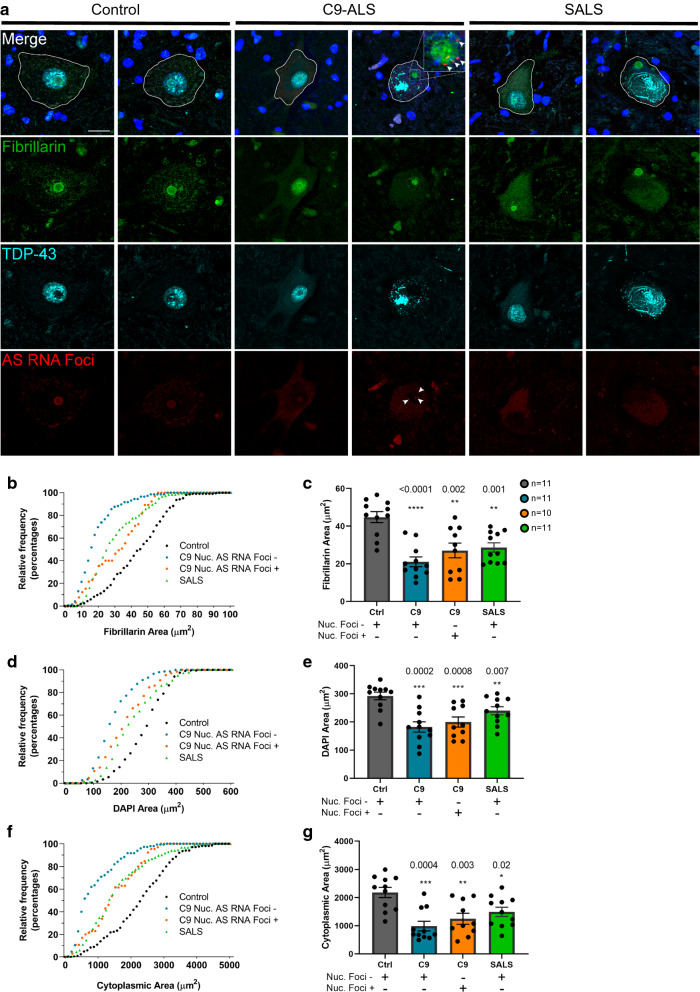
Nucleolar size reduction occurs in neurons with and without antisense RNA foci in C9-ALS. **a** Nucleolar area is identified by fibrillarin staining (green), nuclear area is identified by DAPI and cytoplasmic area is identified by autofluorescence in control, C9-ALS and SALS spinal motor neurons. C-terminal TDP-43 immunofluorescence (cyan) was always nuclear in control neurons and was either nuclear or mislocalized to the cytoplasm in ALS neurons. Antisense RNA foci are identified by FISH staining (red) (nucleolar antisense RNA foci are show in the insert, arrowheads). **b**–**g** Nucleolar area (**b**, **c**), nuclear area (**d**, **e**) and cytoplasmic area (**f**, **g**) are decreased in C9-ALS and SALS neurons with or without nucleolar antisense RNA foci compared to controls

We wanted to see how the presence of antisense RNA foci and TDP-43 mislocalization correlated with reduced nucleolar size in ALS SMNs. Cooper-Knock et al. have previously shown that antisense RNA foci correlate with TDP-43 pathology in SMNs [10]. We further expanded on this finding by showing that the nucleolar localization of antisense RNA foci in cells accounted for 97% of the co-occurrence of antisense RNA foci and TDP-43 mislocalization [1]. In light of these previous observations, we first analyzed our current data, stratifying neurons by the presence of antisense RNA foci (Fig. 3a, Additional file 6: Table S1). While nucleolar, nuclear and cytoplasmic area reductions were seen both in neurons with and without antisense RNA foci (all *p* values < 0.001) (Additional file 5: Fig. S5a-f), we found nucleolar shrinkage was greatest in neurons without nucleolar antisense RNA foci (nucleolar  foci− *p* < 0.0001; nucleolar  foci+ *p* = 0.002) (Fig. 3b, c). Similarly, nuclear and cytoplasmic area were decreased in neurons with or without nucleolar antisense RNA foci (all *p* values < 0.003) (Fig. 3d–g). Nucleolar, nuclear and cytoplasmic area were largely proportional in both control and ALS cases. Thus, it appears that nucleolar shrinkage may occur before pathological markers appear.

### Nucleolar size reduction occurs in neurons with and without TDP-43 mislocalization

**Fig. 4:**
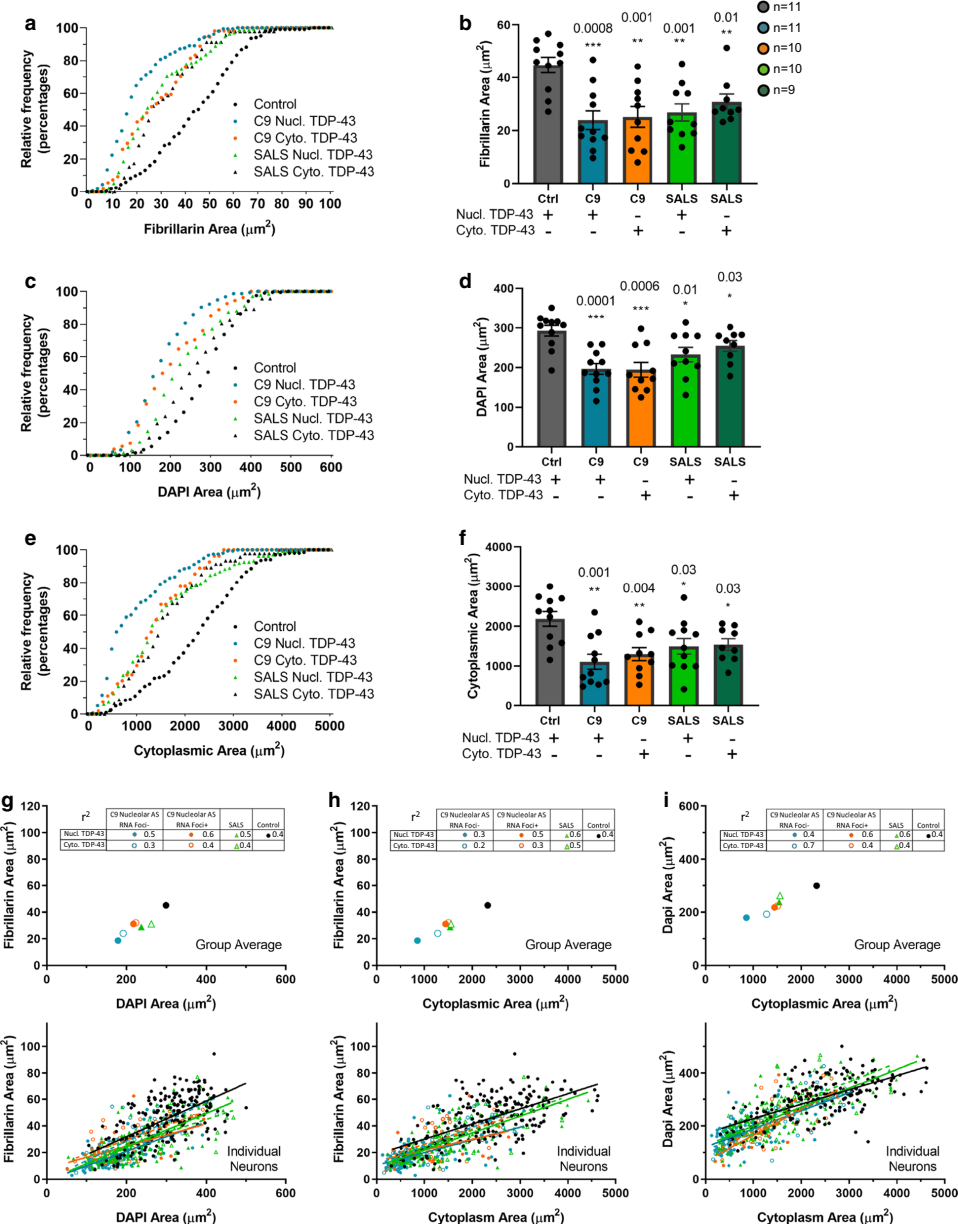
Nucleolar size reduction occurs in neurons with and without TDP-43 mislocalization. **a**–**f** Nucleolar area (**a**, **b**), nuclear area (**c**, **d**) and cytoplasmic area (**e**, **f**) are decreased in C9-ALS with or without TDP-43 mislocalization and in SALS neurons compared to controls. **g**–**i** Scatterplots of group average (top) and individual neurons (bottom) comparing nucleolar and nuclear area (**g**), nucleolar and cytoplasmic area (**h**) and nuclear and cytoplasmic area (**i**) (lines indicate linear regression)

Mizielinska et al., had previously reported that decreased nucleolar volume in C9-FTLD frontal cortical neurons was most prominent in neurons lacking sense RNA foci, poly(GA) or poly(GR) [36]. Since we now also found that nucleolar area was decreased not only in C9-ALS SMNs but also in SALS SMNs, we wondered if other pathological markers such as TDP-43 mislocalization correlated to a greater reduction in nucleolar size. We analyzed our data by stratifying neurons with normal nuclear TDP-43 localization or mislocalized nuclear TDP-43 that formed cytoplasmic aggregates. As expected, using an antibody specific to the C-terminus of TDP-43, we observed that all C9-ALS and SALS SMNs lacking nuclear TDP-43 had cytoplasmic TDP-43 inclusions (Fig. 3a, Additional file 6: Table S2). We found that nucleolar area was reduced both in C9-ALS and SALS SMNs with either nuclear TDP-43 or mislocalized TDP-43 (Fig. 4a, b). However, as with antisense RNA foci, reductions in nucleolar, nuclear and cytoplasmic area were greatest in neurons without TDP-43 mislocalization in both C9-ALS and SALS SMNs (all *p* values < 0.03) (Fig. 4a–f). This further supports that nucleolar shrinkage may precede the appearance of pathological markers.

Lastly, we stratified neurons by the presence of either TDP-43 mislocalization or nucleolar antisense RNA foci or both and compared the correlation of nucleolar, nuclear and cytoplasmic area between these groups. SMNs from controls had the largest nucleoli, nuclei and cytoplasms (Fig. 4g–i). SMNs from SALS cases had smaller nucleoli, nuclei and cytoplasms than controls and this difference was greatest in neurons that had normal nuclear TDP-43 (Fig. 4g–i). SMNs from C9-ALS cases had smaller nucleoli than both controls and SALS and this difference was greatest in neurons with normal nuclear TDP-43 and no nucleolar antisense RNA foci. C9-ALS SMNs with TDP-43 mislocalization and no nucleolar antisense RNA foci had smaller nucleoli, nuclei and cytoplasms than neurons with nucleolar antisense RNA foci with or without TDP-43 mislocalization (Fig. 4g–i). This also suggests that nucleolar stress as marked by nucleolar shrinkage may occur early in disease and precede pathology, either TDP-43 mislocalization and/or antisense RNA foci.

## Discussion

In this study, we expand upon previous findings identifying nucleolar stress in C9-ALS SMNs and extend them to SALS. Overall, SMNs in C9-ALS have smaller nucleoli compared to controls. Surprisingly, this shrinkage occurs in seemingly healthy neurons as defined by absence of pathological markers—TDP-43 mislocalization or antisense RNA foci. This is further supported by the morphometric data, where we found nucleolar shrinkage to occur in the large uniformly sized alpha motor neurons in C9-ALS, which we separated out for analysis from alpha motor neurons that might be shrinking in the process of degeneration and from gamma motor neurons, which we defined by way of molecular and morphometric study. Thus, nucleolar stress as manifested by nucleolar shrinkage appears to be an upstream change, before many of the main molecular and structural changes. While this may seem counterintuitive—it being logical to assume that nucleolar shrinkage progresses as a part of pathological changes within degenerating neurons—in fact, Mizielinska et al. had similarly found nucleolar shrinkage in C9-FTLD frontal cortical neurons to be greatest in neurons without pathological markers, in their case sense RNA foci or sense-encoded DPRs [36]. Thus, at least two studies now have shown consistent findings of nucleolar shrinkage in the absence of pathological markers such as TDP-43 mislocalization/aggregation, RNA foci or DPRs in C9-ALS.

It seems possible that TDP-43 mislocalization, RNA foci or DPRs may drive nucleolar stress before they become grossly visible aggregates and that the methods to determine them are not sensitive enough to detect their presence in the early prodromal state. For example, repeat-containing RNA transcripts could be toxic before forming detectable RNA foci. Similarly, DPRs could be toxic before forming large detectable aggregates. Notably, White et al. have demonstrated that high concentrations of soluble arginine-rich DPRs competitively sequester nucleophosmin and ribosomal RNA in vitro, resulting in disruption of the nucleolar structure [59]. Poly(GR)-induced dispersion of nucleoli is consistent with Mizielinska et al. reporting that poly(GR) correlates with enlarged nucleoli in C9-FTLD frontal cortical neurons [36, 59] even though nucleolar size was smaller overall in C9-ALS. These and our findings could be explained by soluble RNAs or proteins, either poly(GR) or TDP-43, inducing nucleolar shrinkage in the prodromal state and then inducing size increases as they aggregate and become insoluble. Such biophysical states would not likely be detectable by current neuropathological methods. But they could underlie the biphasic nature of nucleolar stress that has been observed in neuropathology. Previously, we have shown that TDP-43 mislocalization is intrinsically linked to nucleolar antisense RNA foci [1]. In rare cases of C9-ALS, it has been shown that TDP-43 pathology occurs later in disease while sense and antisense RNA foci can precede TDP-43 pathology [57]. In the spinal cord, DPRs are rare and increased presence of nucleolar antisense but not sense RNA foci correlate with TDP-43 mislocalization [1, 45]. Thus, it remains possible that repeat expanded RNA transcripts, arginine-rich DPRs and TDP-43 first induce toxicity by way of the nucleolus when they are soluble.

**Fig. 5:**
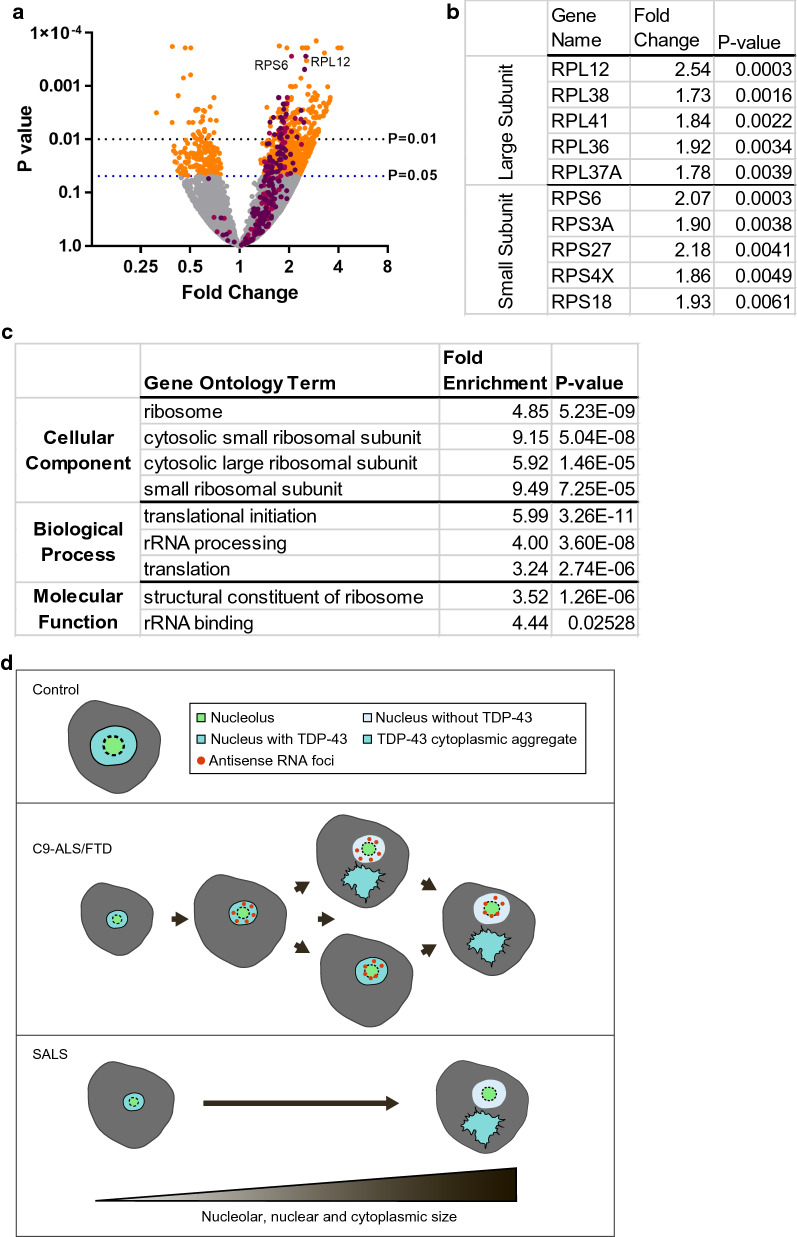
Ribosomal protein mRNA are upregulated in SALS spinal motor neurons. **a** Volcano plot of differential gene expression from laser capture microdissection of SALS spinal motor neurons compared to controls. Significance level, *p* = 0.05 and *p* = 0.01 are marked with blue and black lines, respectively. Large (dark purple) and small (light purple) ribosomal protein subunit genes are denoted (pseudogenes are included). **b** Fold change are shown for the top 5 differentially expressed large or small ribosomal protein subunit mRNAs. **c** Fold enrichment for significantly enriched gene ontology terms relating to ribosomal biogenesis and protein translation. **d** Schematic representation of nucleolar stress preceding antisense RNA foci and TDP-43 mislocalization in C9-ALS and SALS spinal motor neurons

Surprisingly, we also found nucleolar shrinkage in motor neurons in SALS and thus our observations about nucleolar stress in C9-ALS also may apply to SALS. Nucleolar shrinkage was clearly shown to be independent of the main pathological marker, TDP-43 mislocalization, and less clearly but still suggestively by the morphometric data analyzing large uniformly sized alpha motor neurons. The morphometric results do not stand up to multiple testing as well as the C9-ALS data, but since neuropathology is fundamentally an observational science, it along with the TDP-43 mislocalization data and the C9-ALS data is strongly suggestive [43]. Thus, it also seems that nucleolar stress as manifested by nucleolar shrinkage appears to be an upstream change, before many of the main molecular and structural changes, in SALS. Disease pathways including nuclear cytoplasmic transport defects, dysfunctional stress granule assembly, impaired protein clearance and aberrant immune responses have all been shown to contribute to neurodegeneration and it remains to be seen how nucleolar shrinkage and nucleolar stress correlate with these and participate in pathogenesis [2, 6–8, 13, 14, 17, 20–22, 25, 27–29, 31, 47–50, 55, 56, 64, 65, 67]. In this regard, re-analysis of laser capture microdissection data from SALS spinal motor neurons, previously published in Krach et al., revealed nearly uniform upregulation of ribosomal protein mRNA and enrichment of multiple gene ontology terms relating to ribosomal biogenesis and protein translation (Fig. 5a–c) [24]. Similarly, RNA profiling from patient lymphoblasts and motor cortex suggest dysregulation of ribosomal biogenesis also occurs in C9-ALS, which is in line with nucleolar stress marked by a reduction in nucleolar volume [16, 24]. Ribosomal biogenesis is an energy-demanding task and can be inhibited as a cellular response to stress [4, 36]. Alternatively, gain of function mechanisms underlying C9-ALS and SALS can directly impair ribosomal biogenesis. Reduced ribosomal biogenesis and protein translation as well as altered nucleolar size have been shown to occur in cellular models of C9-ALS supporting that C9-ALS gain of toxicity mechanisms disrupt nucleolar function [16, 26, 36, 54, 58, 59]. Thus, nucleolar stress is one of the earliest pathogenic changes in ALS (Fig. 5d).

With the increasing knowledge on how various genetic and environmental factors affect the risk and progression of ALS, it is important to factor in categorization based on sex. Sexual dimorphisms have been reported in human spinal cord; axons in the lateral corticospinal tract were found to be larger in males compared to females [66]. In animals, Mierzejewska-Krzyzowska et al. reported that male rats had larger alpha motor neurons compared to females but Dukkipati et al. found alpha motor neuron size to be similar in wild-type mice [12, 34]. Interestingly, Dukkipati et al. reported that male G93A mice had larger soma size compared to wild-type mice, but female G93A mice had smaller alpha motor neurons compared to wild-types. Sexually dimorphic vulnerability in ALS has been suggested to occur in humans and animal models of disease with males having earlier disease onset, higher incidence and higher prevalence [12, 33, 51]. Our study was inadvertently skewed to include more males than females yet in controls, alpha motor neurons were larger in males than in females. Interestingly, C9-ALS and SALS males and females had similar alpha motor neuron size, however, compared to controls, decrease in neuron size was greater in males than in females. A larger study is needed to investigate the extent to which sexual dimorphism might affect neurodegeneration in ALS, but thus far, several studies in humans and rodents have indicated that males have increased vulnerability in ALS [33, 51, 66]. Integrating sex-specific guidelines and strategies in treatments may prove useful in improving the design and outcomes of clinical trials as well as patient responses to treatments.

It is unknown if reduced nucleolar size occurs in other cell types that display TDP-43 pathology. Of particular interest are glial cells and gamma motor neurons which reside in close proximity to alpha motor neurons. Similarly, large sensory neurons in the posterior horn show TDP-43 pathology but like gamma motor neurons are resilient to neurodegeneration. It is also unknown if reduced nucleolar size occurs in additional cell-types selectively vulnerable to neurodegeneration such as von Economo neurons (VENs) which support social behavior and emotionality and reside in layer V of the anterior cingulate cortex and frontoinsular—the primary regions to degenerate at the onset of FTD [38, 44]. Nuclear clearing and cytoplasmic inclusions of TDP-43 were found to correlate with dystrophy of neurites in frontoinsular VENs but it remains to be seen if nucleolar stress is also a neuropathological feature of VENs [38]. Also, of interest is understanding if nucleolar stress occurs in ALS lacking TDP-43 pathology such as SOD-1 and FUS cases. Nucleolar stress has previously been observed in a broad range of neurodegenerative disease including Alzheimer’s disease, Parkinson’s disease and Huntington’s disease, it is important to unravel if nucleolar stress is a general feature of neurodegeneration and where it lays in the disease process [reviewed in 18].

In summary, we have demonstrated that nucleolar stress occurs in both C9-ALS and SALS spinal motor neurons and is independent of many pathological changes including TDP-43 mislocalization or antisense RNA foci and overall cell shrinkage and is therefore upstream in pathogenesis. This supports and extends previous findings from neuropathology and cellular models and has important implications for pathobiology of ALS.

## Supplementary Information

**Additional file 1. Figure S1:** Nucleolar to cytoplasmic and nuclear to cytoplasmic area ratios in control, C9-ALS and SALS SMNs. **a**, **b** Nucleolar to nuclear area ratio were decreased in C9-ALS and SALS neurons compared to controls. **c**, **d** Nucleolar to cytoplasmic area ratio is not significantly different in C9-ALS or SALS SMNs compared to controls. **e**, **f** Nuclear to cytoplasmic area ratio is significantly increased in C9-ALS but not SALS SMNs compared to controls.

**Additional file 2. Figure S2:** Random sampling of spinal motor neurons for statistical analysis. **a** Number of spinal motor neurons quantified from each patient. **b**–**g** Random sampling of neurons for analyzing difference in nucleolar area (**b**, **c**), nuclear area (**d**, **e**) and cytoplasmic area (**f**, **g**) between control and C9-ALS or SALS neurons.

**Additional file 3. Figure S3:** Analysis of nuclear and nucleolar abnormalities in a sub-sample of spinal motor neurons. **a**–**e** Stratification of immunofluorescence data.** a** Distribution of cytoplasmic area in a sub-sample of control, C9-ALS and SALS alpha motor neurons (cytoplasmic area threshold at 2000 µm^2^). Fibrillarin to cytoplasmic area ratio (**b**) and fibrillarin to nuclear area ratio (**c**) are significantly different in a sub-sample of similarly sized C9-ALS but not SALS neurons compared to controls. Nuclear area (**d**) and nuclear to cytoplasmic area ratio (**e**) are not significantly different in a sub-sample of similarly sized C9-ALS or SALS neurons compared to controls.

**Additional file 4. Figure S4:** Sexual dimorphism in spinal motor neuron nucleolar and nuclear area is abolished in C9-ALS and SALS. **a**, **b** Nucleolar (**a**) and nuclear area (**b**) are significantly larger in male control cases compared to females but are similar in C9-ALS and SALS cases. **c** Cytoplasmic area is not significantly different between male and female in control, C9-ALS or SALS cases. **d**–**i** Correlation between nucleolar (**d**, **g**), nuclear (**e**, **h**) or cytoplasmic area (**f**, **i**) and age at disease onset or disease course, respectively.

**Additional file 5. Figure S5:** Nucleolar size reduction occurs in neurons with and without nucleolar antisense RNA foci in C9-ALS. **a**–**f** Nucleolar area (**a**, **b**), nuclear area (**c**, **d**) and cytoplasmic area (**e**, **f**) are decreased in C9-ALS and SALS neurons with or without antisense RNA foci compared to controls.

**Additional file 6. Table S1:** Nucleolar Antisense (AS) RNA Foci Stratification.** Table S2** TDP-43 Stratification

## Data Availability

The datasets used and/or analyzed during the current study available from the corresponding author on reasonable request.
