# Supplementary figures and images for: Nucleolar stress in C9orf72 and sporadic ALS spinal motor neurons precedes TDP-43 mislocalization
Source: Acta Neuropathol Commun. 2021 Feb 15;9:26. doi: 10.1186/s40478-021-01125-6 (PMC7885352; doi:10.1186/s40478-021-01125-6)

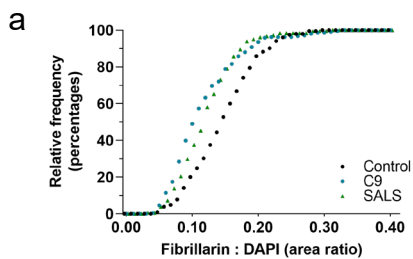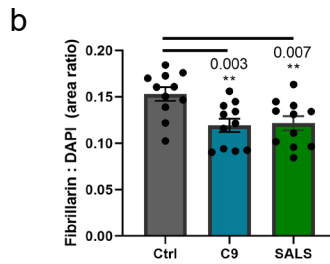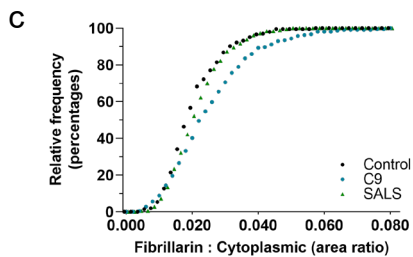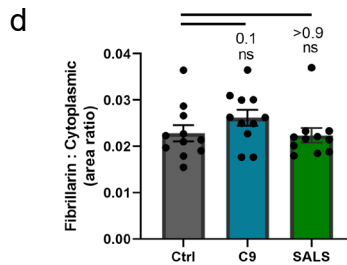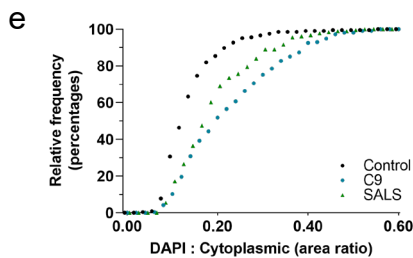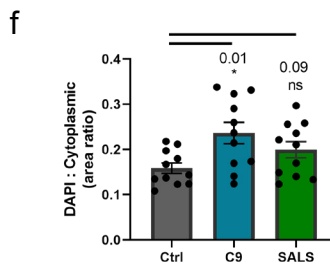

Supplement: Supplementary file 1 — Additional file 1. Figure S1: Nucleolar to cytoplasmic and nuclear to cytoplasmic area ratios in control, C9-ALS and SALS SMNs. a, b Nucleolar to nuclear area ratio were decreased in C9-ALS and SALS neurons compared to controls. c, d Nucleolar to cytoplasmic area ratio is not significantly different in C9-ALS or SALS SMNs compared to controls. e, f Nuclear to cytoplasmic area ratio is significantly increased in C9-ALS but not SALS SMNs compared to controls. [file 40478_2021_1125_MOESM1_ESM.pdf]

a

Number of neurons quantified from each patient

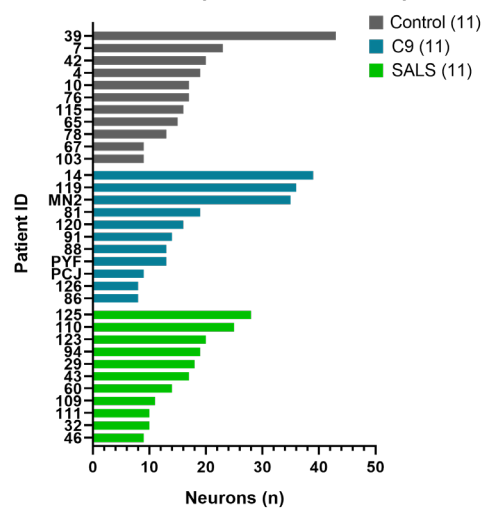

b

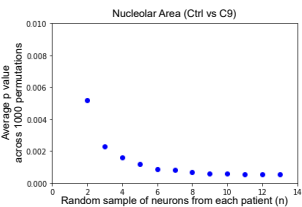

c

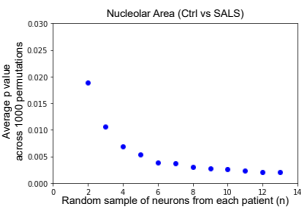

d

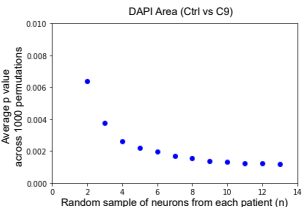

e

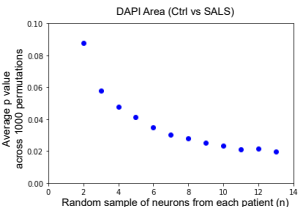

f

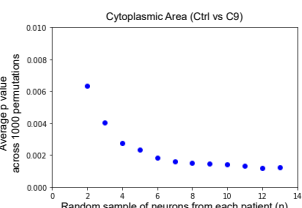

g

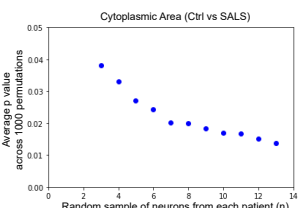

Supplement: Supplementary file 2 — Additional file 2. Figure S2: Random sampling of spinal motor neurons for statistical analysis. a Number of spinal motor neurons quantified from each patient. b–g Random sampling of neurons for analyzing difference in nucleolar area (b, c), nuclear area (d, e) and cytoplasmic area (f, g) between control and C9-ALS or SALS neurons. [file 40478_2021_1125_MOESM2_ESM.pdf]

**a**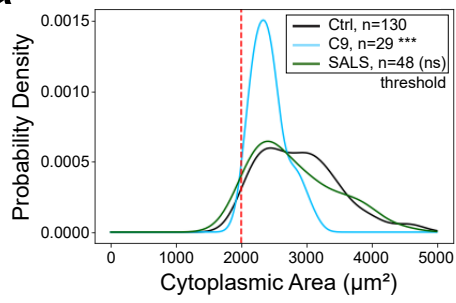**b**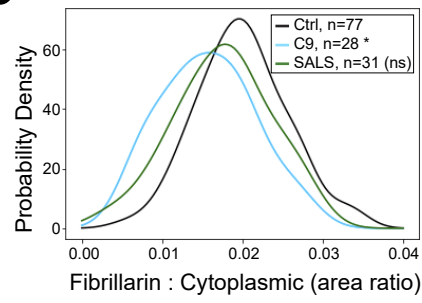**c**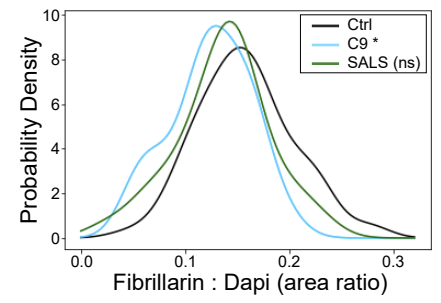**d**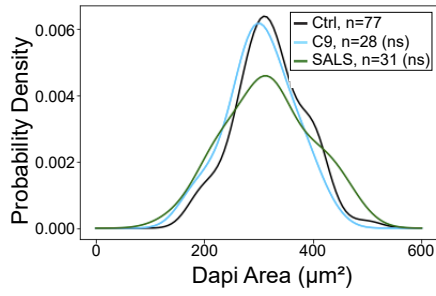**e**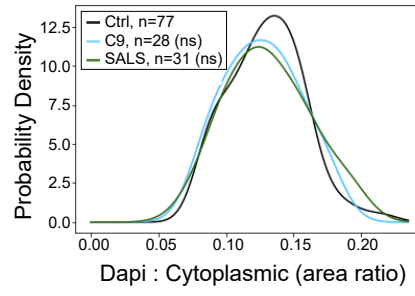

Supplement: Supplementary file 3 — Additional file 3. Figure S3: Analysis of nuclear and nucleolar abnormalities in a sub-sample of spinal motor neurons. a–e Stratification of immunofluorescence data. a Distribution of cytoplasmic area in a sub-sample of control, C9-ALS and SALS alpha motor neurons (cytoplasmic area threshold at 2000 µm2). Fibrillarin to cytoplasmic area ratio (b) and fibrillarin to nuclear area ratio (c) are significantly different in a sub-sample of similarly sized C9-ALS but not SALS neurons compared to controls. Nuclear area (d) and nuclear to cytoplasmic area ratio (e) are not significantly different in a sub-sample of similarly sized C9-ALS or SALS neurons compared to controls. [file 40478_2021_1125_MOESM3_ESM.pdf]

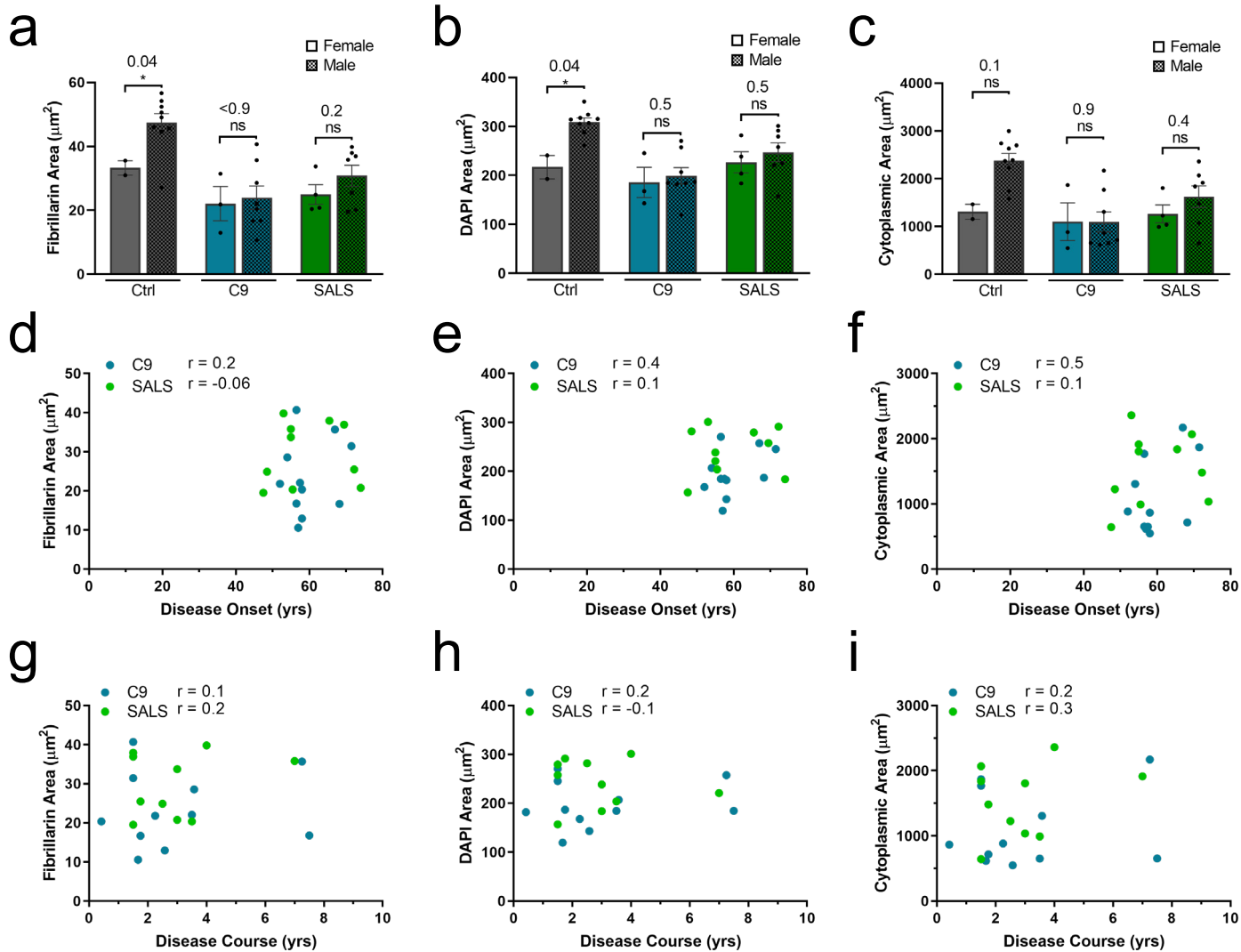

Supplement: Supplementary file 4 — Additional file 4. Figure S4: Sexual dimorphism in spinal motor neuron nucleolar and nuclear area is abolished in C9-ALS and SALS. a, b Nucleolar (a) and nuclear area (b) are significantly larger in male control cases compared to females but are similar in C9-ALS and SALS cases. c Cytoplasmic area is not significantly different between male and female in control, C9-ALS or SALS cases. d–i Correlation between nucleolar (d, g), nuclear (e, h) or cytoplasmic area (f, i) and age at disease onset or disease course, respectively. [file 40478_2021_1125_MOESM4_ESM.pdf]

a

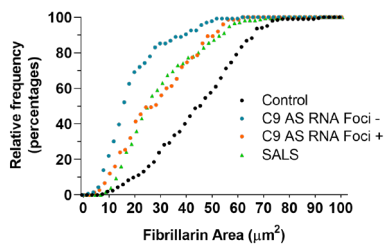

b

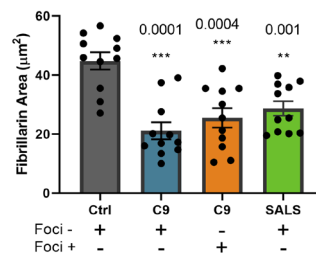

c

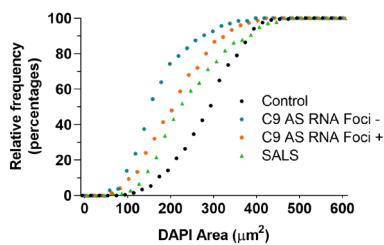

d

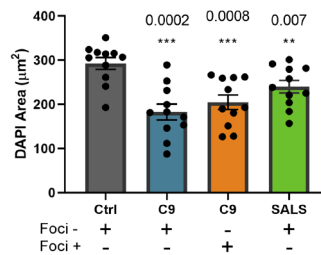

e

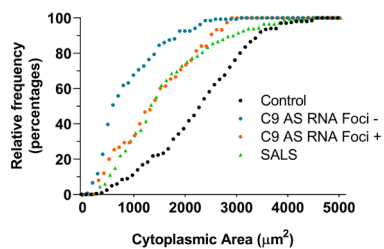

f

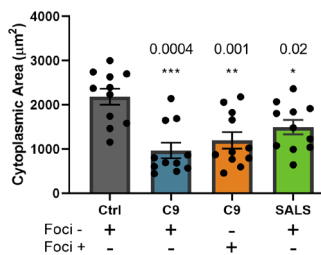

Supplement: Supplementary file 5 — Additional file 5. Figure S5: Nucleolar size reduction occurs in neurons with and without nucleolar antisense RNA foci in C9-ALS. a–f Nucleolar area (a, b), nuclear area (c, d) and cytoplasmic area (e, f) are decreased in C9-ALS and SALS neurons with or without antisense RNA foci compared to controls. [file 40478_2021_1125_MOESM5_ESM.pdf]
