# Supplementary material for: Nucleolar stress in C9orf72 and sporadic ALS spinal motor neurons precedes TDP-43 mislocalization
Source: Acta Neuropathol Commun. 2021 Feb 15;9:26. doi: 10.1186/s40478-021-01125-6 (PMC7885352; doi:10.1186/s40478-021-01125-6)
Supplement: Supplementary file 6 — Additional file 6. Table S1: Nucleolar Antisense (AS) RNA Foci Stratification. Table S2 TDP-43 Stratification [file 40478_2021_1125_MOESM6_ESM.docx]

Supplementary Table 1 Nucleolar antisense (AS) RNA foci Stratification ****Supplementary Table 2 TDP-43 Stratification****
